# More than a cup of tea: an evaluation of an intervention program empowering parents to be effective members of the treating team for young people with eating disorders

**DOI:** 10.1186/2050-2974-2-S1-O35

**Published:** 2014-11-24

**Authors:** Melanie Hunter, Olivia Donaghy, Natalie O'Brien

**Affiliations:** 1 Bayside Child and Youth Mental Health Service, Brisbane, Australia

## 

Bayside Child and Youth Mental Health Service (BCYMHS), a community treatment team in Brisbane, delivers a 6 week 12 hour parent/carer skills based training group for families with a young person affected by an eating disorder based on Dr Janet Treasure's work at the Maudsley hospital (Treasure, Smith and Crane, 2007).

Collaborative care for adolescents has repeatedly been indicated as more effective than individual therapy alone, including parents and carers as a crucial part of the treating team. However, eating disorders often lead to a range of complex behaviours that are confusing and frightening and carers typically display a variety of natural but unhelpful reactions that can inadvertently prolong and entrench eating disorder symptoms and behaviours. Treasure's New Maudsley approach aims to help carers to understand eating disorders, learn communication skills that inhibit the maintenance of the disorder and develop positive coping strategies.

BCYMHS have run 4 groups with a total of 34 carers since 2012. Qualitative outcome measures indicate overwhelmingly positive acceptance and utility of the program, particularly feeling more connected to family members and being aware of strategies to support the young person. Pre and post quantitative measures with parents also indicate change across clinical categories on the DASS. Feedback from clinicians has also indicated the group has increased consistency and collaboration of care across BCYMHS, inpatient wards, and the family.

This abstract was presented in the **Parental Roles in Prevention and Support** stream of the 2014 ANZAED Conference.

